# Association between autoimmune reactions and severity of atopic dermatitis in children with herpes virus infection

**DOI:** 10.1186/1939-4551-6-8

**Published:** 2013-04-29

**Authors:** Pavel Samoylikov, Valentina Gervazieva, Sergey Kozhevnikov

**Affiliations:** 1 Mechnikov Research Institute of Vaccines and Sera, Maliy Kazenniy Pereulok 5a, 103064, Moscow, Russia; 2 Research Institute of Pediatrics and Pediatric Surgery, Taldomskaay 2, 125412, Moscow, Russia

**Keywords:** Atopic dermatitis, IgE- IgG-autoantibodies, Keratin, Elastin, IgG-abs to herpes viruses

## Abstract

**Background:**

Patients with atopic dermatitis (AD) can develop autoantibodies against intracellular proteins. AD patients often suffer from herpes viruses (HV) infection which complicates the inflammatory process in the skin. The aim of the study was to reveal IgE and IgG antibodies (abs) specific to some skin antigens and to compare their levels with the severity of AD with HV infection in children.

**Methods:**

IgE and IgG abs specific to tissue antigens, total IgE, IgE-abs to environmental common allergens as well as IgG abs specific to HV were detected in serum samples by ELISA in 157 AD children.

**Results:**

IgE and IgG antibody production to keratin and elastin was observed in children with AD and elevated proportionally to the severity of AD. IgG – abs to herpes simplex virus was increased in children and associated with the severity of clinical course of AD.

**Conclusion:**

Our data shown that clinical course of severe AD is accompanied with autoimmune response to epidermal antigens (keratin and elastin). Elevated levels of the autoantibodies, especially against the background of HV infection may be useful serological parameter for monitoring of the disease activity.

## Introduction

Atopic dermatitis (AD) is a chronic pruritic inflammatory skin disease that commonly presents during early infancy and childhood but can persist or start in adults. AD is a difficult multiple-factor genetical disease, determined by increased levels of total IgE and specific IgE antibodies (abs) to environmental allergens that are important triggers for this disease [1]. At the same time the autoimmunity to self-related skin tissue antigens has been showed in AD patients [2].

During the past decades several IgE- and IgG-binding autoantigens have been characterized using biochemical and molecular biological methods. IgE-binding autoantigens have been listed in the WHO nomenclature as ara Hom S1-S5 (atopy related antigens Homo sapiens) [3-5]. These autoantigens can activate Type I hypersensitivity reactions (atopy) [6]. IgG-abs to cutaneous tissue antigens (CTA) were also be detected in AD patients [7]. For example, IgG-abs can bind the protein p80 coilin which takes part in RNA processing and transmission of the signal into the cell nucleus [8]. In the skin lens epithelium-derived growth factor (LEDGF/DFS70).

is transported from the nucleus to the cytoplasm of keratinocytes and is accumulated in the keratohyalin granules as the keratinocytes differentiate. DFS70 is the same protein as p75 [9]. Besides, IgE-abs specific to LEDGF/p75 were also detected in AD patients [10].

Although, most autoantigens are intracellular components the damaged tissue in the skin of AD patients might induce the release of autoantigens which then become available to IgE- and probably to IgG antibodies which can aggravate a clinical course of the disease [2]. Patients with AD have a unique predisposition to colonization of the skin by microbial organisms, including herpes viruses (HV) which complicate the course of the inflammatory process in the skin [11] and in addition, have cross-reactivity with cutaneous antigens [12].

The purpose of the present study was to reveal IgE- and IgG-abs specific to some skin antigens and to compare their levels with the severity of AD with herpes virus infection in children.

## Methods

Serum samples from 157 AD patients with the age ranging from 1 to 17 years (88 boys and 69 girls) were tested by ELISA. The AD diagnosis was confirmed using criteria offered by Hanifin and Rajka [13]. All AD patients were divided into two age groups: 103 children of preschool age (from 1 to 6 years, 56 boys and 47 girls) and 54 children of school age (from 7 to 17 years, 32 boys and 22 girls). Based on the severity of the disease patients were grouped as follows: 39 children with mild AD, 55 – moderate AD and 63 – severe AD. Children with mild or moderate AD received treatment in out-patient department, while children with severe AD were treated in the hospital. The severity of AD patients was assessed using the criteria of SCORAD [14]. For control purposes, sera from 29 non-atopic healthy children (from 1 to 17 years, 16 boys and 13 girls) were included. The study has been approved by the Institutional Ethics Committee of Mechnikov Research Institute of Vaccines and Sera (Moscow, Russia) and was performed in accordance with the Helsinki Declaration of 1975.

Serum IgE- and IgG-abs to tissue antigens were determined by ELISA. ELISA plates (Costar, USA) were coated with 100 μl/well of the tissue antigens in concentration 5 μg/ml dissolved in 0.1 M carbonate buffer (pH 9.6) and incubated overnight at 4°C. The plates were washed 2 times with washing buffer (PBS, 0.05% Tween 20) and incubated with 1% bovine serum albumin for 2 h at room temperature (blocking solution). 100 μl/well of patient’s sera diluted in PBS, 0.05% Tween 20 (1:4 for IgE detection; 1:100 for IgG detection) were added and incubated for 1 hour at 37°C. Then the plates were washed 3 times with washing buffer followed by adding 100 μl/well of HRP-conjugated anti-human IgE and IgG detection mouse monoclonal antibodies (Polignost, Russia) diluted 1:1000 for 1 hour at 37°C. Plates were washed 5 times with washing buffer and 100 μl/well of tetramethylbenzidine (Sigma, USA) were added for 20 min at 37°C to develop color reaction which was stopped by the addition of 100 μl/well of 50% H_2_SO_4_. Extinctions (optical densities, 450 nm) were determined with an ELISA reader (Stat Fax - 2100, USA). Optical densities were converted into ME/ml for IgE-abs and into μg/ml for IgG-abs levels, using standard reference reagents (Dr. Fooke, Germany). All determinations were carried out in duplicates. The following CTA: keratin, type III and VI collagen, elastin, myosin (Sigma, USA) were used for ELISA (Table 1).

**Table 1:** Skin distribution of tissue antigens, that were used in the present study

**Antigens**	**Distribution**
Keratin	Keratin filaments are abundant in keratinocytes in the cornified layer of the epidermis
Type III and VI collagen	Intercellular substance of dermis
Elastin	Connective tissue of skin
Myosin	Smooth muscle cells of dermis

The levels of total IgE and specific IgE-abs to environmental common allergens were detected using ImmunoCAP 100® (Phadia, Sweden). Total IgE in kU/l and specific IgE - abs in kUA/l were converted into classes according to the manufacturer’s recommendations: 0 class - less than 0.35 kUA/l; 1 class – from 0.35 to 0.7 kUA/l; 2 class – from 0.7 to 3.5 kUA/l; 3 class – from 3.5 to 17.5 kUA/l; 4 class – from 17.5 to 50 kUA/l; 5 class – from 50 to 100 kUA/l and 6 class – more than 100 kUA/l).

The levels of IgG-abs to herpes simplex virus (HSV), cytomegalovirus (CMV) and Epstein-Barr virus (EBV) were determined using ELISA kits (VECTOR-BEST, Russia), that included mixed HSV1 and HSV2 antigens, recombinant CMV antigen and EBNA-1 p72. The levels of IgG-abs to HSV and EBV were expressed in the units of optical density while abs to CMV in PE/ml (Paul Ehrlich Institute units) in accordance with the manufacturer’s manual.

Serum samples were obtained from each subject (AD patients and healthy controls) by venipuncture. The blood samples were left to clot for up to 60 minutes at room temperature (22°C) and then centrifuged at 3000 rpm for 10 minutes. Sera were stored until use at −20°C.

The distributions of the received data were not normal (in accordance with the Kolmogorov-Smirnov test). For descriptive statistics all data were expressed as median (Me) and quartile range (QR). A nonparametric Mann–Whitney *U*-test was used to compare the levels of total IgE, IgG-abs to herpes viruses, IgE- and IgG-abs to CTA in healthy children and AD patients, as well as between AD patient groups. The correlation between the levels of total IgE and specific IgE-, IgG-abs to tissue proteins was assessed using the Spearman index of co-graduation. All statistical analysis was performed using Microsoft Excel program and StatSoft Statistica 6 for Windows.

## Results

For a serological characterization of the AD disease activity (manifestation of atopy) we determined the levels of IgE-abs specific to food, domestic and opportunistic bacterial allergens and total IgE in serum samples of each subject (AD patients and healthy control). It was shown that food allergens were more etiologically significant in children of pre-school age, while inhalant allergens – in children of school age. For example, 9 children of pre-school age with mild AD demonstrated IgE-abs specific to milk (39%, 1 class), and 6 children of school age – IgE-abs specific to *D. pteronyssinus* and *D. farinae* (37%, 1 class). 17 children of pre-school age with moderate AD also shown IgE-abs specific to milk (41%, 1–3 classes), and 7 children of school age – IgE-abs specific to animal epidermal allergenss (50%, 1–3 classes). Children with severe AD of pre-school and school age shown high levels of IgE-abs specific to milk, egg and house dust mite allergens (1–6 classes).

The levels of total IgE in all AD patients groups were higher than that of healthy subjects of the same age and were increased in proportion to the severity of AD within the range from 1 to 5000 kU/l. The maximal level of total IgE was observed in pre-school age children with severe AD [Me = 360 kU/l (1080)] (Tables 2 and 3).

**Table 2:** The levels and frequency of detection of IgE- and IgG-abs to tissue antigens in children of preschool age with AD

**Severity of AD**	**Amount of patients**	**Levels of total IgE KU/l**	**Antigens**	**Cut-off**		**Level of abs (QR) to tissue antigens**		**Frequency of occurrence of abs, %**	
				**IgE-abs МЕ/ml**	**IgG-abs μg/ml**	**IgE-abs МЕ/ml**	**IgG-abs μg/ml**	**IgE-abs**	**IgG-abs**
**Mild**	23	26.5 (68)	Keratin	1.48	255	1.4 (5.6)*	149.2 (128)*	30.4*	21.7*
			Collagen VI	1.4	73	1.4 (0.6)	67.3 (111)	34.8	43.5
			Elastin	1.4	48	1.4 (1.0)*	25 (16.3)	34.8*	21.7
			Collagen III	1.4	147	1.4 (−)	62.8 (41.9)	21.7	8.7
			Myosin	3.1	154	2.06 (2.4)	53.7 (76.8)	34.8	8.7
**Moderate**	41	107 (275)	Keratin	1.48	255	1.67 (7.8)	260.8 (97.4)	60.0	56.1
			Collagen VI	1.4	73	1.4 (1.2)	64.8 (56.5)	48.8	41.5
			Elastin	1.4	48	1.78 (2.5)	25 (27.1)	53.7	26.8
			Collagen III	1.4	147	1.4 (3.17)	73.7 (88.4)	48.8	19.5
			Myosin	3.1	154	2.88 (9.0)	83 (162.3)	46.3	26.8
**Severe**	39	360 (1080)	Keratin	1.48	255	2.16 (5.58)* **	291.5 (496.6)* **	61.5*	56.4*
			Collagen VI	1.4	73	1.4 (−)	27.2 (26.7)	15.4	15.4
			Elastin	1.4	48	1.81 (0.49)* **	25 (16.9)	53.9*	23.1
			Collagen III	1.4	147	1.4 (−)	25 (40.3)	12.8	5.1
			Myosin	3.1	154	1.4 (−)	40.8 (104.5)	10.3	20.5

* – valid differences in the levels of IgE- and IgG-abs to tissue antigens between mild and severe AD patient groups (p < 0.05).

** – valid increased levels of IgE- and IgG-abs to tissue antigens (p < 0.05).

**Table 3:** The levels and frequency of detection of IgE- and IgG-abs to tissue antigens in children of school age with AD

**Severity of AD**	**Amount of patients**	**Levels of total IgE KU/l**	**Antigens**	**Cut-off**		**Level of abs (QR) to tissue antigens**		**Frequency of occurrence of abs, %**	
				**IgE-abs МЕ/ml**	**IgG-abs μg/ml**	**IgE-abs МЕ/ml**	**IgG-abs μg/ml**	**IgE-abs**	**IgG-abs**
**Mild**	16	19 (36)	Keratin	1.48	206	1.7 (4.68)*	209.8 (213.5)*	50*	68.8*
			Collagen VI	1.4	49	1.4 (1.2)	82.5 (156.1)**	31.3	62.5
			Elastin	1.4	25	1.4 (1.07)*	25 (56.8)	43.8*	31.3
			Collagen III	1.4	54	1.4 (0.71)	45.2 (77.5)	31.3	43.8
			Myosin	3.1	200	1.87 (9.5)	25 (140.5)	43.8	18.8
**Moderate**	14	72 (133)	Keratin	1.48	206	1.87 (1.5)	281.1 (90.5)**	57.1	71.4
			Collagen VI	1.4	49	1.4 (0.05)	59.1 (49.4)	28.6	57.1
			Elastin	1.4	25	1.48 (1.0)	38.3 (104.5)	57.1	50
			Collagen III	1.4	54	1.4 (−)	50.9 (70.8)	21.4	42.9
			Myosin	3.1	200	1.4 (1.92)	136.6 (147)	28.6	21.4
**Severe**	24	128 (471)	Keratin	1.48	206	2.71 (12.29)* **	296.2 (214.1) * **	62.5*	75*
			Collagen VI	1.4	49	1.4 (−)	25 (18.3)	4.2	29.2
			Elastin	1.4	25	2.69 (1.38)* **	25 (10.7)	58.3*	29.17
			Collagen III	1.4	54	1.4 (−)	25 (33.5)	8.3	29.2
			Myosin	3.1	200	1.4 (−)	40.29 (60.2)	8.3	16.7

* – valid differences in the levels of IgE- and IgG-abs to tissue antigens between mild and severe AD patient groups (p < 0.05).

** – valid increased levels of IgE- and IgG-abs to tissue antigens (p < 0.05).

Before evaluation of autoimmune reactions to CTA were detected in the serum samples of healthy subjects. It was shown that in healthy children the levels of IgE-abs to CTA were very low and did not differ between groups of pre-school and school age children. IgE-abs to CTA were as follows: to keratin - below 1.48 ME/ml; to collagen III, collagen VI and elastin - below 1.4 ME/ml; to myosin - below 3.1 ME/ml. The levels of IgG-abs to these antigens were presented in Tables 2 and 3. We accepted these data as a cut-off value (a sum of median levels of these antibodies and quartile range) and all the values of IgG-abs to CTA in AD patient’s groups that were above cut-off value were considered as the elevated autoantibody levels in serum samples.

**Figure 1:**
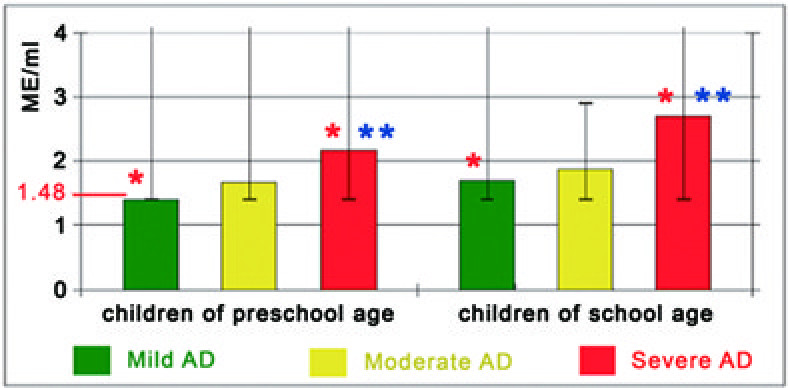
**The levels of IgE-abs to keratin and quartile range against severity of AD.** Normal levels of these abs are under 1,48 ME/ml. * – valid differences in the levels of IgE-abs to keratin between groups. ** – valid increased levels of IgE-abs to keratin.

**Figure 2:**
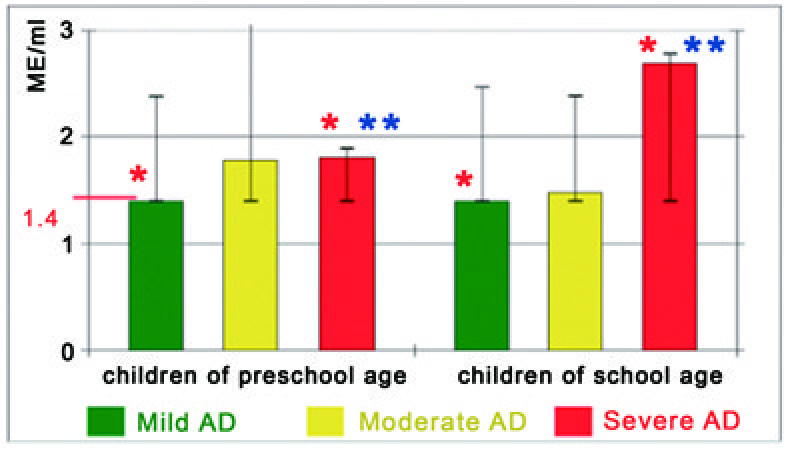
**The levels of IgE-abs to elastin and quartile range against severity of AD.** Normal levels of these abs are under 1,4 ME/ml. * – valid differences in the levels of IgE-abs to elastin between groups. ** – valid increased levels of IgE-abs to elastin.

IgE-abs specific to CTA were detected in the sera of children with AD. It was shown that the levels and the frequency of detection of IgE-abs specific to keratin and elastin were increased in proportion to the severity of AD (Figures 1 and 2 and Tables 2 and 3). The levels of these IgE-abs in sera of severe AD patients were significantly higher (pre-school age children: keratin – 2.16 (5.58) ME/ml and to elastin – 1.81 (0.49) ME/ml; school age children: keratin – 2.71 (12.29) ME/ml), elastin – 2.69 (1.38) ME/ml) in comparison with both mild AD patients (pre-school age: keratin – 1.4 (5.6) ME/ml, elastin – 1.4 (1.0) ME/ml; school age: keratin – 1.7 (4.68) ME/ml), elastin – 1.4 (1.07) ME/ml) and healthy children group (p < 0.05). The levels of IgE-abs to other CTA were normal or insignificantly increased (p > 0.05). The positive correlation was found between the levels of total IgE and IgE-abs specific to keratin in children with severe AD (r = 0.35; p < 0.05).

**Figure 3:**
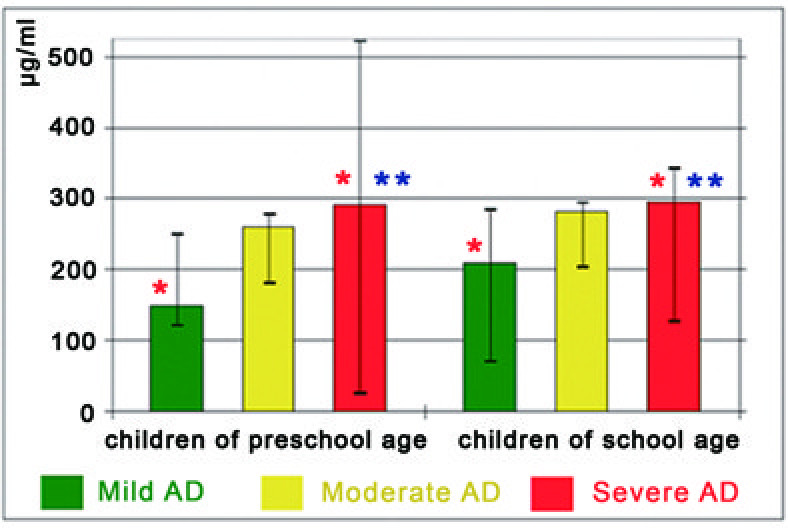
**The levels of IgG-abs to keratin and quartile range against severity of AD.** * – valid differences in the levels of IgG-abs to keratin between groups. ** – valid increased levels of IgG-abs to keratin.

The levels and the frequency of detection of IgG-abs to keratin were also being increased in proportion to the severity of AD (Figure 3 and Tables 2 and 3). In children with severe AD the levels of these abs were also higher (preschool age: keratin – 291.5 (496.6) μg/ml; school age: keratin – 296.2 (214.1) μg/ml), than that of children with both mild AD (preschool age: keratin – 149.2 (128) μg/ml; school age: keratin – 209.8 (213.5) μg/ml) and healthy group (p < 0.05). In addition, we found substantially higher levels of IgG-abs specific to collagen VI in children of school age with mild AD, specific to keratin in children of school age with moderate AD and to keratin in children of pre- and school age with severe AD in compare to healthy children group. There were no significant differences in the levels of IgE and IgG autoantibodies between mild and moderate AD patient groups as well as between moderate and severe AD patient groups.

IgG-abs specific to CMV and EBV were increased in all age groups of patients with AD in comparison with cut-off value (children of preschool age – max CMV Me = 2.43 (5.24) ЕР/ml, max EBV Me =2.59 (0.98); children of school age – max CMV Me = 6.07 (6.13) ЕР/ml, max EBV Me = 2.75 (0.57)). While IgG-abs specific to CMV and EBV in AD patients of all age groups reflected the dynamic of immune response in the population as a whole, the level and the frequency of detection of HSV-IgG-abs were increased in children of school age and associated with severity of clinical course of AD (see Tables 4 and 5).

**Table 4:** The levels and frequency of detection of IgG-abs to herpes viruses in sera of children of preschool age

**Severity of AD**	**Amount of patients**	**Antigens**	**Levels of IgG-abs**		**Frequency of occurrence of IgG-abs in patients, %**
			**Cut-off (in HV-negative people**)	**In children with AD, Me (QR)**	
Mild	23	HSV	0.223	0.169 (1.28)	34.78
		CMV	0.2	0.39* (3.72)	91.3
		EBV	0.45	2.19* (1.37)	95.65
Moderate	41	HSV	0.223	0.173 (2.53)	46.34
		CMV	0.2	1.2* (5.43)	92.68
		EBV	0.45	2.59* (0.98)	92.68
Severe	39	HSV	0.223	0.23 (2.81)	51.28
		CMV	0.2	2.43* (5.24)	89.74
		EBV	0.45	0.86* (1.87)	92.31

The levels of IgG-abs to HSV and EBV are in units of OD. The levels of IgG-abs to CMV are in ЕР/ml.

* – valid differences in the levels of IgG-abs to herpes viruses between children with AD and HV-negative people (p < 0.05).

**Table 5:** The levels and frequency of detection of IgG-abs to herpes viruses in sera of children of school age

**Severity of AD**	**Amount of patients**	**Antigens**	**Levels of IgG-abs**		**Frequency of occurrence of IgG-abs in patients, %**
			**Cut-off (in HV-negative people**)	**In children with AD, Me (QR)**	
Mild	16	HSV	0.223	0.19 (2.68)	43.75
		CMV	0.2	6.07* (6.13)	93.75
		EBV	0.45	2.75* (0.57)	87.5
Moderate	14	HSV	0.223	0.21 (2.65)	50
		CMV	0.2	4.59* (5.67)	92.86
		EBV	0.45	2.63* (1.82)	92.86
Severe	24	HSV	0.223	2.93* (3.01)	70.83
		CMV	0.2	1.35* (5.48)	91.67
		EBV	0.45	2.04* (2.45)	91.67

The levels of IgG-abs to HSV and EBV are in units of OD. The levels of IgG-abs to CMV are in ЕР/ml.

* – valid differences in the levels of IgG-abs to herpes viruses between children with AD and HV-negative people (p < 0.05).

## Discussions

In the present study we have expanded the list of autoantigens which may play pathogenetic role in the development of severe AD. At first for serological characterization of the atopic disease we determined total IgE and specific IgE in serum samples of AD patients and healthy persons. Taking into consideration of a large variety of clinically relevant allergens in the developing of AD we used a total IgE level as some kinds of universal sign of disease activity in comparison with the intensity of autoimmune reactions. It has been showed, that children with mild or moderate AD had much lower levels of total IgE compared to severe AD children [15]. At the same time it is quite understandable that total IgE level is not unique characterization of disease activity. In this connection we decided to explore the development of autoreactivity, in particular, IgE, and IgG antibody response to autoantigens in AD patients with different degrees of disease activity.

In the beginning IgE- and IgG-abs to some endogenous antigens in healthy persons were determined to find cut-off value. Next, we divided all AD patients into two age groups (preschool age and school age) because the levels of IgG-autoantibodies were different in healthy children of those age groups, whereas the levels of IgE-antibodies were very low and were not depended on the age. Establishing the reliable cut-off value gave opportunity to determine the increased levels IgE- and IgG-abs to skin antigens in AD patients. We were shown that AD patients with high levels of IgE- and IgG-reactivity to keratin and elastin may demonstrate much more severe manifestations of the disease.

In addition to that the positive correlation between the levels of total IgE and IgE-abs specific to keratin was observed in children with severe AD (p < 0.05). In our work we used mixed type of keratin which consisted of both intracellular (cytoskeleton of keratinocytes in the epidermis) and extracellular proteins (stratum corneum of the epidermis) and elastin which was an extracellular protein of connective tissue of dermis. These intra- and extracellular protein components of skin can be liberated at the sites of tissue damage and became available to IgE- and IgG-abs. Intracellular IgE-specific autoantigens appear to be released into circulation and occured as IgE immune complexes, which might reach the target organs of AD that could lead to the release of biological mediators through binding to effector cells (e.g. mast cells, basophiles, eosinophils). IgE-mediated presentation of autoallergens may also activate autoreactive T cells to release pro-inflammatory cytokines, inducing those for mediating of the delayed-type hypersensitivity reactions [2].

On the other hand the development of autoimmune responses may be linked to cross-reactive antigens. Some environmental proteins have similar structural homology with human proteins. IgG-abs to P-62 protein (synthetic protein similar to one of EBV antigens) can react with human epidermal keratin, denatured type II collagen and actin [12]. Moreover, herpes viruses can induce apoptosis of CD4^+^CD25^+^Foxp3 Тreg cells by mean of CD95-L interaction with infected dendritic cells [16,17], that might reduce the regulatory function of the immune system. In case of inflammation in the skin it promotes the release of autoantigens and leads to increase of autoantibodies in AD patients. Chronic skin inflammation in AD patients may cause some abnormalities in the activity of lymphocytes that may promote the development of autoimmune reactions. The autoantibodies to skin tissue antigens might not directly involved in pathogenesis of AD but may be the markers of target tissue injury leading to the provocation of an autoimmune response [18].

Our results show that clinical course of the severe AD is accompanied by the autoimmune response to epidermal antigens (keratin and elastin). The detection of the elevated levels of the autoantibodies, especially against the background of herpetic virus infection can improve the diagnostic criteria of AD severity and allow clinicians to choose the most safe and effective treatment of AD patients.

## Competing interests

The authors declare that they have no competing interests.

## Authors’ contributions

PS: adaptation of the ELISA kits to determine serum IgE- and IgG-abs to tissue antigens, carrying out of the tissue autoantibody and HV-IgG-abs studies, participation in the analysis of the data and the preparation of the manuscript. VG: the idea of this study, the leadership of the work, participation in the analysis of the data, discussion of the results and the preparation of the manuscript. SK: carrying out of clinical work, diagnosis, preparation of biomaterial for the study. All authors read and approved the final manuscript.
